# Prenatal (1–3)IGF‐1 Treatment Is Ineffective and Behaviorally Detrimental in a Rat Model of Cortical Malformation

**DOI:** 10.1002/brb3.71405

**Published:** 2026-04-16

**Authors:** Minyoung Lee, Eun‐Jin Kim, Mi‐Sun Yum

**Affiliations:** ^1^ Department of Pediatrics University of Ulsan College of Medicine Seoul South Korea; ^2^ Asan Institute for Life Sciences, Asan Medical Center Seoul South Korea; ^3^ Department of Pediatrics Asan Medical Center Children's Hospital Seoul South Korea

**Keywords:** (1–3)IGF‐1, cognitive function, malformation of cortical development (MCD), methylazoxymethanol acetate (MAM), prenatal intervention, seizure susceptibility

## Abstract

**Background:**

Malformations of cortical development (MCD), a major cause of early‐onset epilepsy, arise from disruptions in fetal corticogenesis, suggesting that prenatal intervention may be required to alter their developmental trajectory. Insulin‐like growth factor‐1 (IGF‐1) has been proposed as a potential therapeutic agent due to its roles in neuronal survival, synaptogenesis, and plasticity. However, whether prenatal or prolonged early‐life IGF‐1 exposure can modify the trajectory of malformed cortical circuits remains unknown.

**Methods:**

Pregnant dams were exposed to MAM to induce cortical malformation, and fetal brain development was assessed at embryonic day 19 (E19) using in utero MRI. Offspring were assigned to receive prenatal and/or prolonged early postnatal administration of the truncated IGF‐1 analog (1–3)IGF‐1. Outcomes after IGF‐1 treatment were evaluated across multiple domains, including brain morphology, body growth, seizure susceptibility, neurometabolic profiles (^1^H‐MRS), and behavioral performance (Y‐maze, open field, fear conditioning).

**Results:**

Fetal MRI revealed significantly reduced brain size in MAM‐exposed fetuses, indicating that structural abnormalities were already established prenatally. Neither prenatal nor prolonged postnatal (1–3)IGF‐1 treatment improved whole‐brain growth, neurometabolite levels, or susceptibility to N‐methyl‐D‐aspartate‐induced spasms. Moreover, long‐term IGF‐1 exposure led to adverse behavioral outcomes, including impaired spatial working memory in the Y‐maze and increased freezing during conditioning, without affecting locomotor activity or fear recall. No beneficial effects were observed across any treatment paradigm.

**Conclusion:**

Although IGF‐1 has known neurotrophic properties, early and sustained exposure to the (1–3)IGF‐1 analog did not ameliorate structural‐, metabolic‐, or seizure‐related abnormalities and produced detrimental behavioral effects in this model of cortical malformation. These findings underscore the importance of developmental timing and molecular specificity when applying IGF‐1‐based interventions and caution against the assumption that earlier or prolonged treatment is necessarily beneficial in malformed cortical circuits.

## Graphical abstract

Prenatal MAM exposure induces cortical malformation with reduced brain size evident on fetal MRI (E19). (1–3)IGF‐1 treatment showed no rescue at P15 and was associated with long‐term behavioral impairment, including reduced novelty preference and increased freezing behavior.

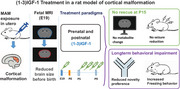

## 1 Introduction

Malformations of cortical development (MCD) are a major cause of early‐onset, drug‐resistant epilepsy and developmental delay in children. These neurodevelopmental disorders arise from disruptions in critical processes such as neuronal proliferation, migration, or cortical organization during fetal life (Juric‐Sekhar and Hevner 2019; Khandelwal et al. 2022). Because the majority of cortical neurons are generated and positioned during fetal development, the key pathogenic events underlying MCD occur before birth, making prenatal intervention biologically plausible. Despite advances in prenatal imaging that enable early detection of cortical malformations, no disease‐modifying or curative treatments are currently available. Most children with MCD rely instead on symptomatic management, particularly antiseizure medications, which offer limited efficacy and do not alter the underlying pathology.

To address this therapeutic gap, preclinical studies have employed the methylazoxymethanol acetate (MAM)–induced rat model, which reliably recapitulates key features of MCD, including cortical thinning, structural disorganization, and heightened seizure susceptibility (Kim et al., 2017). Previous studies in this model have shown that early postnatal administration of insulin‐like growth factor 1 (IGF‐1) enhances synaptic protein expression and may reduce seizure burden, suggesting a neuroprotective role for IGF‐1 in immature, malformed brains (Lee et al., 2023).

IGF‐1 plays a critical role in brain development by promoting neuronal survival, differentiation, synaptogenesis, and myelination (D'Ercole and Ye, 2008; Tropea et al., 2009). In rodent models, IGF‐1 administration during early postnatal periods enhances dendritic arborization and increases expression of synaptic proteins such as PSD‐95 and synaptophysin (Tropea et al., 2009). These actions are particularly relevant in the context of cortical malformations, where impaired synaptic connectivity is a core pathological feature (Lee et al., 2020). The MAM model, in particular, demonstrates aberrant GABAergic signaling and delayed synaptic maturation, both of which may be amenable to early IGF‐1 intervention (Lee et al., 2020).

The (1–3)IGF‐1 fragment, derived from the N‐terminal tripeptide of IGF‐1, is thought to retain neurotrophic efficacy while minimizing mitogenic risk. Preclinical studies in Rett syndrome and other models of neurodevelopmental disorders suggest that (1–3)IGF‐1 can cross the blood–brain barrier and modulate excitatory/inhibitory balance, enhance synaptic plasticity, and improve behavioral outcomes (Abbas et al., 2024; Keam, 2023; Vahdatpour et al., 2016). However, its effects in malformed cortical circuits remain unknown. Furthermore, the timing and duration of IGF‐1‐based treatments in relation to critical neurodevelopmental windows have not been systematically evaluated. Understanding whether prenatal or sustained IGF‐1 exposure can modify the structural or functional trajectory of the malformed brain is essential for developing effective interventions.

Because MCD originates during fetal corticogenesis, it is essential to understand the extent to which structural abnormalities are already present at the time of prenatal intervention. To capture these early developmental alterations, we incorporated fetal magnetic resonance (MR) imaging to quantify fetal brain size at embryonic day 19 (E19). This approach allowed us to assess whether MAM‐induced cortical malformation is detectable in utero and to contextualize the timing of prenatal (1–3)IGF‐1 administration relative to ongoing structural abnormalities.

In this study, we investigated whether early (1–3)IGF‐1‐based interventions could improve neurodevelopmental outcomes in a rat model of MCD. Using the MAM model, we systematically evaluated the effects of prenatal and prolonged postnatal administration of N‐terminal tripeptide analog (1–3)IGF‐1. We assessed outcomes across multiple domains, including brain morphology, seizure susceptibility, neurometabolic profiles via in vivo magnetic resonance spectroscopy (MRS), and cognitive and behavioral performance. By targeting early developmental windows with IGF‐1‐based treatment, we aimed to determine whether such interventions could reverse or mitigate the structural and functional consequences of cortical malformation.

## 2 Methods

### 2.1 Experimental Animals and MAM‐Induced MCD Model

**FIGURE 1:**
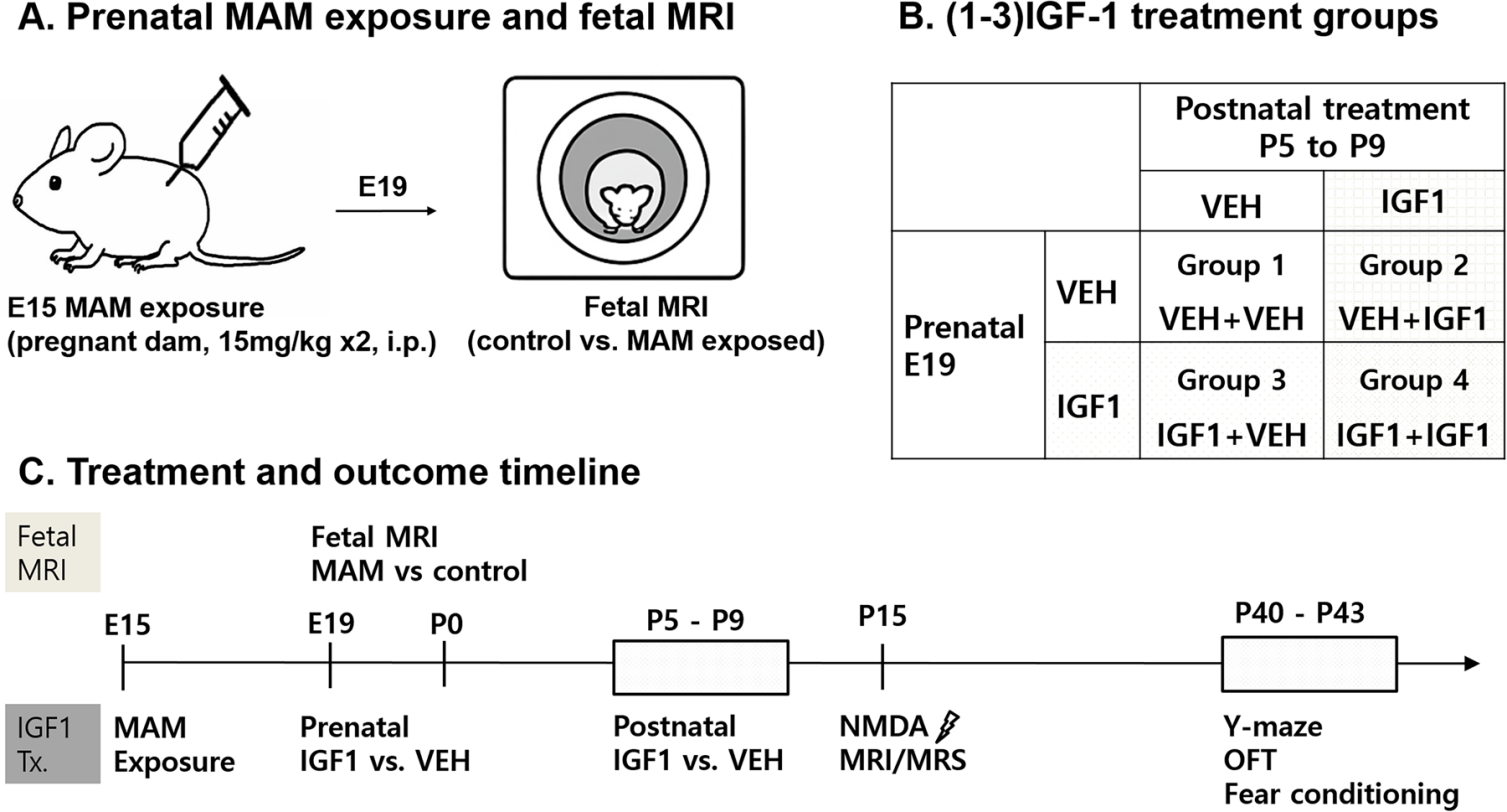
Experimental design for the fetal MRI cohort and the IGF‐1 treatment cohort. (A) Pregnant dams received methylazoxymethanol acetate (MAM: 15 mg/kg, twice, i.p.) on embryonic day 15 (E15). Fetal MRI was performed on E19 to assess baseline brain size in control and MAM‐exposed fetuses. (B) Four IGF‐1 treatment groups were generated in an independent cohort using a 2 × 2 design based on prenatal (E19) and postnatal (P5–P9) administration of (1–3)IGF‐1 or vehicle: Group 1: VEH + VEH, Group 2: VEH + IGF, Group 3: IGF + VEH, and Group 4: IGF + IGF. (C) Timeline of experimental procedures. The upper timeline depicts the fetal MRI cohort, showing MAM exposure at E15 followed by fetal MRI at E19. The lower timeline depicts the IGF‐1 treatment cohort, showing prenatal IGF‐1 or vehicle administration at E19, postnatal IGF‐1 or vehicle treatment from P5 to P9, MRI/MRS and NMDA‐induced spasms at P15, and behavioral testing for Group 1 and Group 4 (Y‐maze, open field test, and fear conditioning) at P40–P43.

Experiments were approved by the Institutional Animal Care and Use Committee of the Ulsan University College of Medicine and conformed to the Revised Guide for the Care and Use of Laboratory Animals (8th Edition, 2011). To investigate the therapeutic potential of IGF‐1‐based interventions in malformation of cortical development (MCD), we employed the MAM rat model. Pregnant Sprague‐Dawley dams (Orient Bio Inc., Korea) were injected intraperitoneally with 15 mg/kg MAM (MRIGlobal, USA) twice on embryonic day 15 (E15), at 8:00 a.m. and 6:00 p.m., to induce MCD in the offspring; control dams received the same volume of saline (Figure 1).

To assess the presence of fetal brain malformation at the time of prenatal treatment, fetal MRI was performed on embryonic day 19 (E19) in MAM‐exposed dams (*N* = 8) and control dams (*N* = 7). All dams were housed under a 12‐h light/dark cycle with ad libitum access to food and water. The day of birth was designated as postnatal day 0 (P0).

Sex‐balanced offspring were randomly assigned to treatment groups, and the sex distribution is provided in Table S1. For all experiments, each animal contributed one data point; thus, the number of observations corresponds directly to the number of animals included in each analysis. Offspring from multiple litters were used in each experimental group. To minimize potential litter effects, pups were randomly selected and distributed across treatment groups so that no group consisted predominantly of animals from a single litter.

### 2.2 Fetal MRI Acquisition and Brain Area Measurement

Fetal MRI was performed using a 9.4T/160 mm small‐animal MRI system (Agilent Technologies, Santa Clara, CA, USA) equipped with a 72‐mm quadrature volume coil. Pregnant dams were anesthetized with 2.0%–2.5% isoflurane in a 1:2 mixture of O_2_ and N_2_O. Body temperature was maintained at 37.5 ± 0.5°C using a controlled air‐heating system, and respiratory rate was continuously monitored throughout image acquisition.

T2‐weighted images were acquired using a fast spin‐echo (FSE) sequence. Coronal images were obtained with the following parameters: repetition time (TR) = 4000 ms, effective echo time (TE) = 30.43 ms, echo train length (ETL) = 8, bandwidth = 50 kHz, echo spacing = 7.61 ms, number of excitations (NEX) = 2, 20 contiguous slices of 1.5 mm thickness, no inter‐slice gap, and slice angulation perpendicular to the axial plane. Axial images were acquired with TR = 4000 ms, TE = 38.59 ms, ETL = 8, bandwidth = 50 kHz, echo spacing = 9.65 ms, NEX = 4, 20 contiguous slices of 0.5 mm thickness, no inter‐slice gap, and slice angulation perpendicular to the coronal plane. Respiratory gating was applied during image acquisition.

Raw data were reconstructed using VnmrJ software (version 4.0; Agilent Technologies). Cartesian *k*‐space sampling was used. No additional filtering was applied. Motion correction was not required due to stable anesthesia and minimal fetal movement. Signal intensity normalization was performed to compensate for coil sensitivity variations.

**FIGURE 2:**
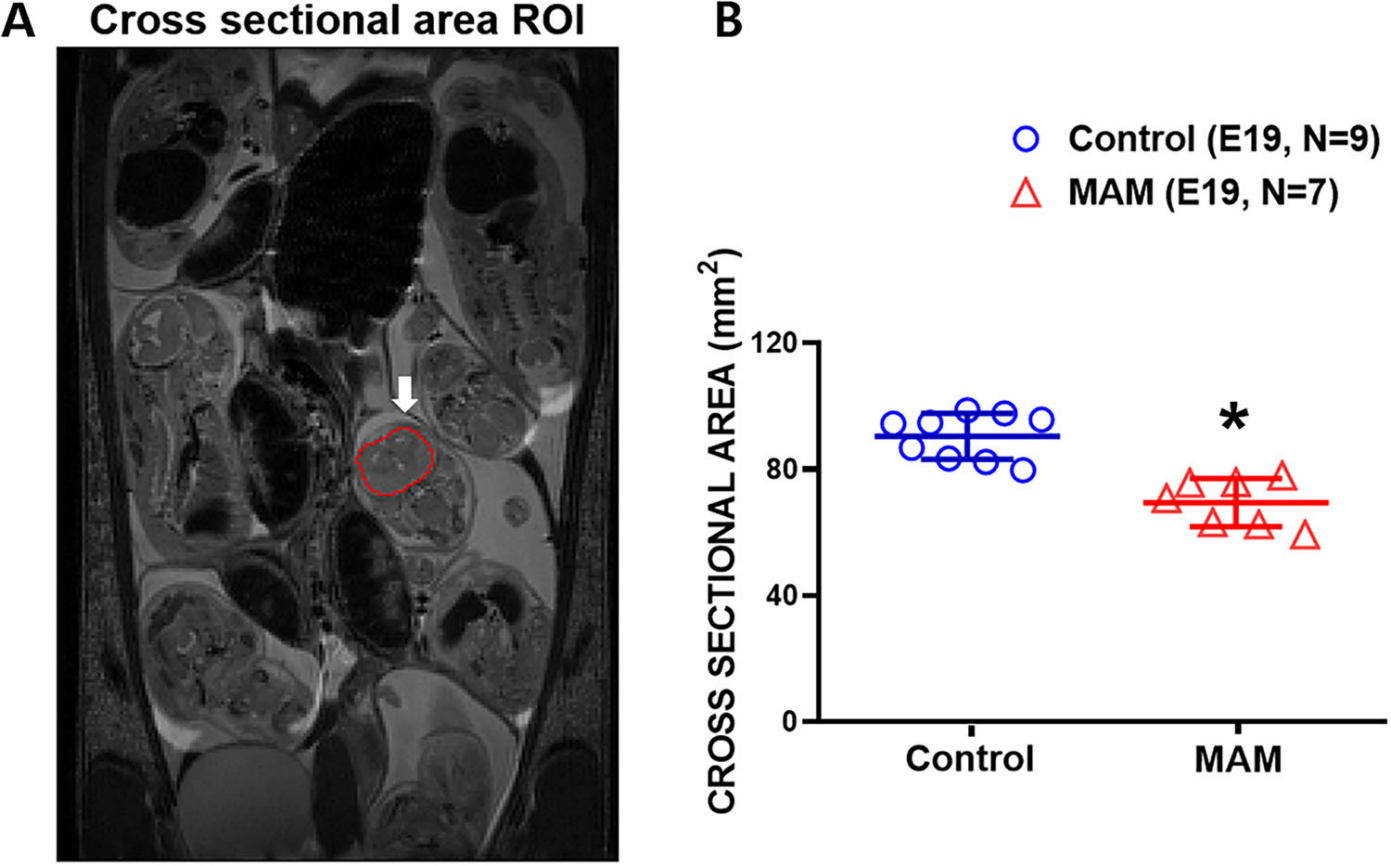
Reduced fetal brain cross‐sectional area in MAM‐exposed embryos at E19. (A) Representative coronal fetal MRI image at embryonic day 19 (E19). The fetal brain region of interest (ROI) is outlined in red and indicated by an arrow. (B) Quantification of fetal brain cross‐sectional area measured on MRI. MAM‐exposed embryos (red triangles, *n* = 7) showed significantly smaller cross‐sectional brain area compared with control embryos (blue circles, *n* = 9) (Mann–Whitney *U* test, *U* = 63.0, *p* < 0.001). Data are presented as mean ± standard deviation. *significant difference among groups.

For quantitative analysis, we selected a consistent coronal plane for each fetus based on established anatomical landmarks used in fetal rodent MRI. Specifically, we identified the slice where the lateral ventricles, hippocampal primordium, and hypothalamus were simultaneously visible (Figure 2A). This level provides a reproducible rostrocaudal reference across embryos and minimizes variability due to fetal orientation. A region of interest outlining the entire fetal brain was manually drawn on this plane, and cross‐sectional brain area was calculated.

### 2.3 (1–3) IGF‐1 Administration Protocol

To evaluate prenatal and postnatal effects of (1–3)IGF‐1, we administered 10 mg/kg, a dose previously reported to exert synaptic and behavioral modulation in neurodevelopmental disorder models, particularly Rett syndrome, without overt mitogenic adverse effects (Abbas et al., 2024; Tropea et al., 2009; Vahdatpour et al., 2016; (Figure S1).

Litters were assigned to four treatment combinations based on prenatal (E19) and postnatal (P5–P9) administration. All (1–3)IGF‐1 injections were delivered intraperitoneally in sterile saline containing 0.1% BSA (Figure 1B). For prenatal treatment, (1–3)IGF‐1 or vehicle was administered on embryonic day 19 (E19) by intraperitoneal injection to the pregnant dam (maternal intraperitoneal injection).

- Group 1 (VEH + VEH): Prenatal vehicle at E19; postnatal vehicle (P5–P9).
- Group 2 (VEH + IGF‐1): Prenatal vehicle at E19; postnatal IGF‐1 (10 mg/kg/day, P5–P9).
- Group 3 (IGF‐1 + VEH): Prenatal IGF‐1 at E19; postnatal vehicle (P5–P9).
- Group 4 (IGF‐1+IGF‐1): Prenatal IGF‐1 at E19; postnatal IGF‐1 (10 mg/kg/day, P5–P9).

### 2.4 NMDA‐Induced Spasms at P15

To evaluate seizure susceptibility, rats received a single intraperitoneal injection of N‐methyl‐D‐aspartic acid (NMDA; 15 mg/kg, Sigma‐Aldrich) at P15. Animals were observed for 90 min postinjection by observers blinded to treatment group allocation. The number of spasms, latency to initial tail twisting (“tailing”), and latency to first spasm were recorded. Body weight was measured on P15 to assess treatment‐related growth effects.

### 2.5 Behavioral Testing

Behavioral testing was conducted between postnatal day 40 (P40) and P43 in animals from paradigm 3, within a standardized testing environment during the light phase (8:00 a.m.–8:00 p.m.). All behavioral assessments were conducted by experimenters blinded to treatment group.

Tests were administered in the following order:

- Y‐maze novelty preference (NP) test (P40): Working memory was assessed in a two‐phase Y‐maze paradigm. After a 5‐min familiarization trial with two open arms, rats were reintroduced for a 5‐min test phase with access to all three arms. The NP index was calculated as:
$$\mathrm{NP}\;\mathrm{index}=\left(\frac{\mathrm{novel}\;\mathrm{arm}\;\mathrm{entries}}{\mathrm{novel}+\mathrm{familiar}\;\mathrm{arm}\;\mathrm{entries}}\right)\times \;100$$
- Open field test (P41): To assess spontaneous locomotor activity, each rat was placed in a 60 cm × 60 cm black arena for 5 min. Rest time, movement velocity, and zone‐specific movement (central vs. peripheral) were analyzed using a computerized tracking system (SMART 3.0; Panlab S.L.U., Spain).
- Fear conditioning (P42–P43): Contextual and cued fear responses were measured following tone–shock pairing (P42) and reexposure sessions in contexts (P42) and cued stimuli (P43). Freezing behavior was quantified via an automated load‐cell detection system (Panlab S.L.U.).

### 2.6 In Vivo MR Imaging and MRS

**FIGURE 3:**
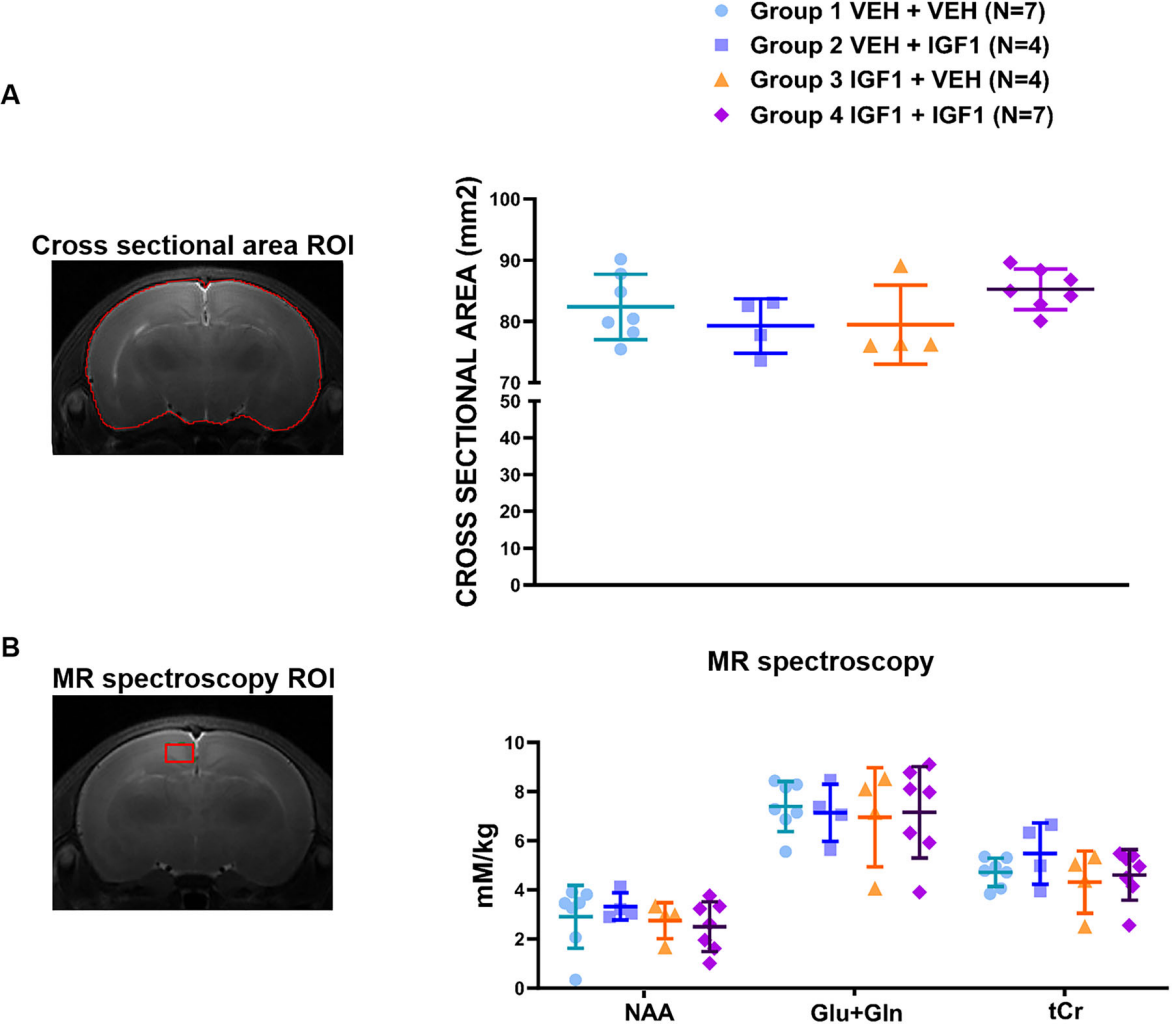
Effects of long‐term (1–3)IGF‐1 treatment on brain morphology and neurometabolic profiles at P15. (A) Whole‐brain area measurements were obtained from a representative coronal section located 2.0 mm posterior to the bregma. MAM‐exposed rats receiving prenatal and/or postnatal (1–3)IGF‐1 treatment showed no significant difference in whole‐brain area among the four groups (Kruskal–Wallis test, *H* = 4.887, *p* = 0.180, Group 1: *n* = 7, Group 2: *n* = 4, Group 3: *n* = 4, Group 4: *n* = 7). (B) In vivo 1H‐MRS analysis of the cingulate cortex showed no significant group differences in N‐acetylaspartate (NAA), glutamate + glutamine (Glu + Gln), or total creatine (tCr). Data are presented as mean ± SD.

To assess both structural brain development and neurometabolic profiles, in vivo proton MRS was performed at P15 in a subset of animals. Whole‐brain cross‐sectional area was measured on a single coronal T2‐weighted slice located 2.0 mm posterior to bregma to assess gross brain size (Figure 3A).

In vivo proton MRS was performed at P15 using a 7.0T/160‐mm Bruker PharmaScan system (Bruker BioSpin, Ettlingen, Germany) equipped with a 72‐mm transmit volume coil and a mouse brain surface receiver coil. Animals were anesthetized with 1% isoflurane in a 1:2 mixture of O_2_ and N_2_O, with physiological parameters continuously monitored throughout acquisition. Magnetic field homogeneity was optimized using the FASTMAP shimming method. Water suppression was achieved using the VAPOR technique. The receiver bandwidth was set to 5 kHz. Spectra were acquired using a point‐resolved spectroscopy (PRESS) sequence (TR/TE = 5000/13.4 ms; 128 averages) with a voxel (1.5 × 1 × 4 mm^3^) positioned in the right cingulate cortex (bregma +1.2 to −2.8 mm). Data acquisition and reconstruction were performed using Paravision 6.0.1 software. Metabolite concentrations—including glutamate, γ‐aminobutyric acid (GABA), N‐acetylaspartate (NAA), creatine, myo‐inositol, and taurine—were quantified using LCModel (version 6.0; RRID:SCR_014455) and normalized to tissue water content.

### 2.7 Statistical Analysis

All data are presented as mean ± standard deviation (SD). Statistical analyses were performed using SPSS Statistics 22.0 (IBM Corp.; RRID:SCR_002865). For comparisons of continuous variables across multiple groups, data distributions and variance homogeneity were inspected to guide the choice between parametric and nonparametric tests. Seizure‐related measures were analyzed using one‐way analysis of variance (ANOVA), followed, when appropriate, by Tukey's honest significant difference (HSD) test for post hoc comparisons.

Due to small sample sizes and uncertain distributional assumptions, brain size, body weight, and neurometabolic measures obtained from MRS were analyzed using the Kruskal–Wallis test, with Mann–Whitney *U* tests applied for pairwise comparisons when indicated. For two‐group comparisons, including fetal brain measurements at E19 and behavioral assays where only two treatment groups were compared, statistical differences were assessed using the Mann–Whitney *U* test. A *p*‐value < 0.05 was considered statistically significant.

## 3 Results

### 3.1 Reduced Fetal Brain Size on Embryonic Day 19 (E19)

Fetal MRI performed at E19 demonstrated that MAM exposure at E15 resulted in a significant reduction in overall brain size. Compared with controls, MAM‐exposed fetuses showed visibly smaller brain contours and decreased cortical thickness on coronal sections, particularly along the dorsal pallial region. Quantitative measurements of the whole‐brain area confirmed a robust reduction in brain size in the MAM group. Mean fetal brain area was 88.89 ± 6.81 in controls (*n* = 9) and 69.45 ± 7.59 in MAM‐exposed fetuses (*n* = 7) (Mann–Whitney *U* test, *U* = 63.0, *p* < 0.001; rank‐biserial correlation = −1.0) (Figure 2). These findings indicate that cortical malformation is already apparent during late gestation and establish that MAM induces early structural deficits prior to birth, providing a baseline for subsequent postnatal assessments of growth and treatment response.

### 3.2 Effects of Long‐Term (1–3)IGF‐1 on Brain Structure, Body Weight, Neurometabolism, and Seizure Susceptibility at P15

Prenatal and postnatal administration of (1–3)IGF‐1 did not affect cross‐sectional brain area at P15. Mean cross‐sectional brain area was 82.39 ± 5.34 mm^2^ in Group 1 (*n* = 7), 79.26 ± 4.45 mm^2^ in Group 2 (*n* = 4), 79.48 ± 6.44 mm^2^ in Group 3 (*n* = 4), and 85.26 ± 3.30 mm^2^ in Group 4 (*n* = 7) (Kruskal–Wallis analysis *H* = 4.887, *p* = 0.180) (Figure 3A).

In vivo MRS at P15 similarly revealed no significant differences among treatment groups in NAA (group means: 2.90 ± 1.82, 3.32 ± 0.55, 2.75 ± 0.73, 2.50 ± 1.01; *p* = 0.225), glutamate + glutamine (Glu + Gln; 7.39 ± 1.02, 7.14 ± 1.16, 6.96 ± 2.01, 7.16 ± 1.86; *p* = 0.949), or total creatine (tCr; 4.71 ± 0.58, 5.48 ± 1.25, 4.32 ± 1.27, 4.60 ± 1.03; *p* = 0.655) (Figure 3B).

**FIGURE 4:**
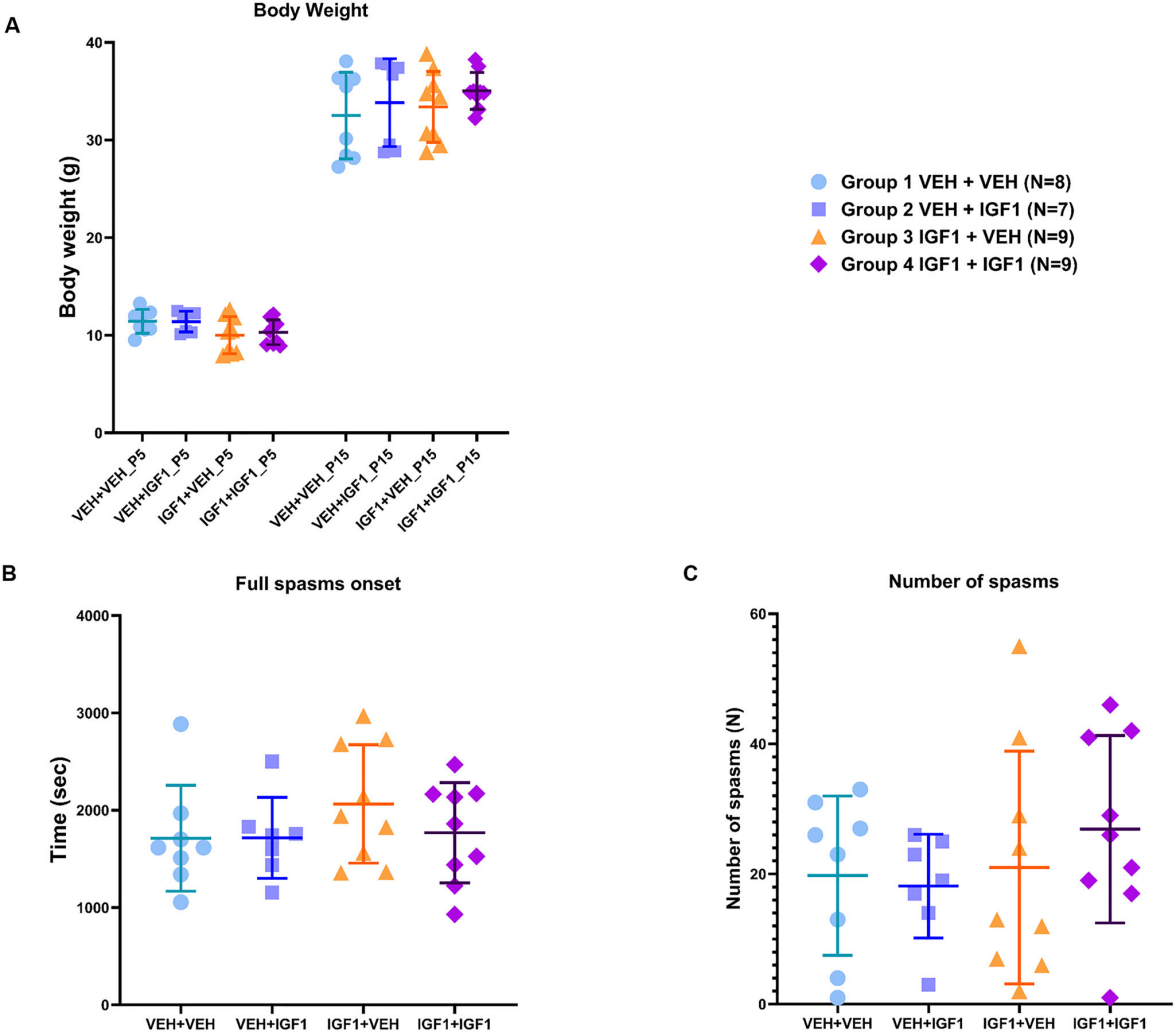
Effects of long‐term (1–3)IGF‐1 treatment on body weight and seizure susceptibility in MAM‐exposed rats. (A) There was no significant change in body weight across four treatment groups at P5 and P15. Seizure susceptibility was assessed by measuring (B) latency to full spasms, and (C) total number of NMDA‐induced spasms. One‐way ANOVA revealed no significant differences in full spasm latency (*F*(3, 29) = 0.861, *p* = 0.472), or total spasm number (*F*(3, 29) = 0.626, *p* = 0.604, Group 1: *n* = 8, Group 2: *n* = 7, Group 3: *n* = 9, Group 4: *n* = 9). Data are presented as mean ± SD.

Body weight also did not differ among groups at P5 (group means: 11.44 ± 1.22 g, 11.40 ± 1.07 g, 10.02 ± 1.90 g, 10.31 ± 1.27 g; *H* = 5.775, *p* = 0.123) or P15 (32.52 ± 4.43 g, 33.83 ± 4.50 g, 33.40 ± 3.62 g, 35.05 ± 1.88 g; *H* = 1.420, *p* = 0.701) (Figure 4A). Seizure susceptibility was also comparable across treatment conditions. Mean latency to first spasm was 1256.13 ± 147.56 s, 1384.14 ± 225.15 s, 1610.33 ± 497.29 s, and 1322.67 ± 359.25 s for Groups 1–4, respectively (*F*(3,29) = 1.726, *p* = 0.184). Mean latency to full spasms was 1711.25 ± 544.13 s, 1717.00 ± 415.99 s, 2064.44 ± 608.31 s, and 1768.67 ± 514.63 s (*F*(3,29) = 0.861, *p* = 0.472) (Figure 4B), and the total number of spasms was 19.75 ± 12.24, 18.14 ± 7.97, 21.00 ± 17.87, and 26.89 ± 14.41 (*F*(3,29) = 0.626, *p* = 0.604) (Group 1: *n* = 8, Group 2: *n* = 7, Group 3: *n* = 9, Group 4: *n* = 9) (Figure 4C).

### 3.3 Effects of Long‐Term (1–3)IGF‐1 Treatment on Behavior

To evaluate the broader neurodevelopmental effects of IGF‐1‐based treatments, behavioral assessments were performed exclusively in animals receiving long‐term IGF‐1 treatment and VEH + VEH controls.

**FIGURE 5:**
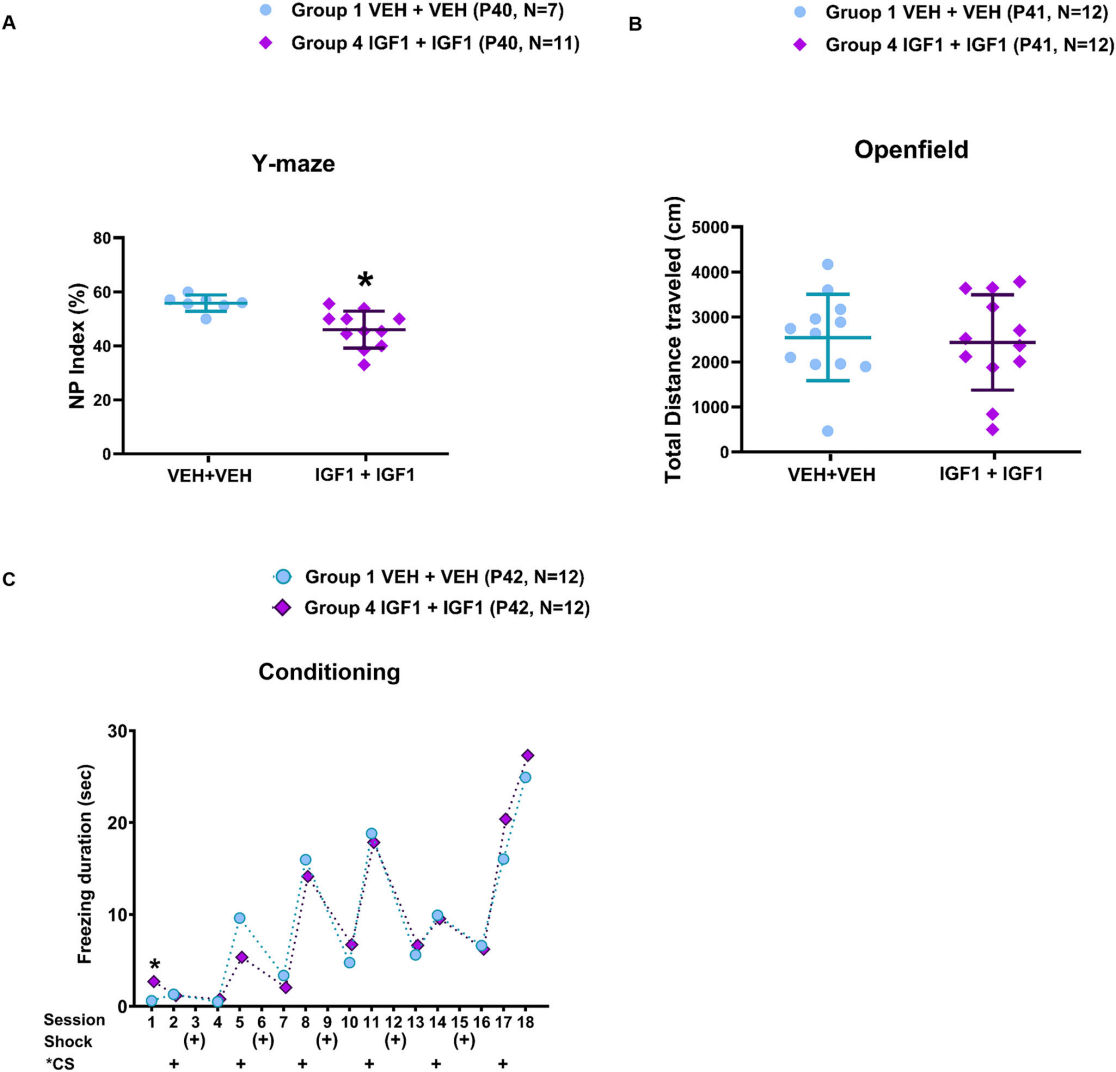
Effects of long‐term (1–3)IGF‐1 treatment on behavior in MAM‐exposed rats. (A) Y‐maze testing at P40 revealed a significant reduction in novelty preference index in IGF‐1 + IGF‐1‐treated rats compared to VEH + VEH controls, indicating impaired spatial working memory (Mann–Whitney *U* = 5.000, *p* = 0.002, *n* = 7 vs. *n* = 11). *p < 0.05. (B) Open field test at P41 showed no significant differences in total distance traveled (*p* = 0.954, VEH + VEH: *n* = 12 vs. IGF1 + IGF1: *n* = 12), suggesting intact locomotor function. CS: conditioned stimuli. *p < 0.05. (C) During fear conditioning at P42–P43, freezing duration in the initial training session was significantly increased in the long‐term IGF‐1 group (*U* = 39.000, *p* = 0.042, VEH + VEH: *n* = 12 vs. IGF1 + IGF1: *n* = 12), while no significant group differences were observed in contextual or cued fear responses. Data are presented as mean ± SD.

Y‐maze testing at P40 revealed a significant reduction in NP index in rats receiving long‐term (1–3)IGF‐1 treatment. The mean NP index was 55.81 ± 3.03 in VEH + VEH controls (*n* = 7) and 46.02 ± 6.82 in IGF‐1 + IGF‐1‐treated rats (*n* = 11) (Mann–Whitney *U* = 5.000, *p* = 0.002) (Figure 5A), indicating impaired spatial working memory.

In the open field test at P41, total distance traveled did not differ significantly between groups (2543.59 ± 959.57 cm vs. 2434.58 ± 1058.83 cm, *p* = 0.954). Time spent in the central zone was also comparable (484.08 ± 290.51 s vs. 367.00 ± 221.89 s, *p* = 0.356; VEH + VEH: *n* = 12, IGF‐1 + IGF‐1: *n* = 12) (Figure 5B), suggesting preserved locomotor activity and anxiety‐related behavior.

During fear conditioning (P42–P43), freezing duration during the initial training session was significantly increased in the long‐term IGF‐1 group (2.68 ± 3.56 s) compared to controls (0.60 ± 1.29 s) (Mann–Whitney *U* = 39.000, *p* = 0.042; *n* = 12 per group). However, no significant differences were observed in contextual or cued fear recall (Figure 5C).

## 4 Discussion

In this study, we assessed fetal brain abnormalities induced by MAM exposure to determine whether structural deficits were already detectable at E19, when fetal brain anatomy can be reliably visualized by MRI. We then examined whether prenatal and/or early postnatal long‐term administration of the truncated IGF‐1 analog (1–3)IGF‐1 (Bach, 2018) could ameliorate neurodevelopmental deficits in a rat model of MCD. Contrary to our expectations based on the known neurotrophic properties of IGF‐1 (Liu et al., 1993) and our prior reports (Lee et al., 2023), which demonstrated its beneficial effects when administered during later postnatal periods, neither prenatal nor prolonged early postnatal (1–3)IGF‐1 treatment improved brain or body growth, seizure susceptibility, or neurometabolic status in MAM‐exposed offspring (Figures 3 and 4). Moreover, long‐term IGF‐1 exposure was associated with adverse behavioral outcomes, including impaired spatial working memory in the juvenile period (Figure 5). These findings suggest that the therapeutic efficacy of IGF‐1‐based interventions may critically depend on the timing and specific IGF‐1 isoform used, and that early, prolonged exposure to truncated analogs may be ineffective—or even detrimental—in the context of MCD.

Previously, we demonstrated the presence of early postnatal brain malformations in MAM‐exposed animals (Lee et al., 2024). In humans and other mammals, the early fetal period represents a critical window for neocortical development: most cortical neurons are generated prenatally, and many have already migrated to their appropriate cortical layers and begun forming essential brain networks by mid‐gestation (Stiles and Jernigan, 2010; Subramanian et al., 2020). These developmental features suggest that the most effective window for intervention in cortical malformations is likely prenatal, before the major phases of neuronal proliferation and migration have concluded.

Moreover, advances in fetal neuroimaging now allow reliable detection of MCD using fetal MRI (Ganapathy et al., 2025), raising the possibility that therapeutic strategies may be applied even before birth. To evaluate the degree of fetal brain malformation in our model, we adopted in utero fetal brain imaging as previously described (Deans et al., 2008) and were able to successfully quantify fetal brain size, indicating that a clear size difference was already present in the fetal period. Early postnatal rodent brain development overlaps substantially with late‐gestation human brain maturation, underscoring the importance of careful cross‐species timing interpretation (Clancy et al., 2007; Semple et al., 2013).

Although IGF‐1 has been shown to promote synaptogenesis and plasticity in normal and injured brains (Littlejohn et al., 2021; O'Kusky and Ye, 2012), its effectiveness in the MCD context appears limited in this study. The lack of improvement in seizure susceptibility across all treatment paradigms suggests that the disrupted neural architecture in MAM‐exposed brains at very early developmental stages may be insufficiently responsive to IGF‐1‐mediated modulation. A prior study demonstrated that rhIGF‐1 enhances the expression of synaptic markers and reduces seizure burden when administered postnatally in the same model (Lee et al., 2023). In addition, (1–3)IGF‐1 has shown potential therapeutic effects in a tetrodotoxin (TTX)‐induced murine model of epileptic spasms (Ballester‐Rosado et al., 2022). However, in the present study, even prenatal or combined treatment regimens failed to alter seizure outcomes, suggesting a potential ceiling effect or mismatched developmental timing. Another study (Song et al., 2016) also showed that neuroprotective levels of exogenous IGF‐1 increased epileptic activity, suggesting that the stage of the disease is a critical factor influencing the neuroprotective efficacy of IGF‐1.

These observations raise the possibility that factors beyond dose or duration may limit the responsiveness of malformed cortical circuits to IGF‐1‐based interventions. One possible explanation for the lack of therapeutic efficacy is that the malformed cortical circuits induced by MAM exposure may be intrinsically resistant to trophic modulation at the developmental stage examined. MAM administration at E15 disrupts neuronal proliferation and migration during a critical window of corticogenesis, leading to permanent alterations in laminar organization and network architecture (Kim et al., 2017; Lee et al., 2024; Subramanian et al., 2020). By E19, as demonstrated by our fetal MRI findings, structural abnormalities are already established. Although IGF‐1 is known to enhance synaptic plasticity, dendritic maturation, and neuronal survival (D'Ercole and Ye, 2008; O'Kusky and Ye, 2012; Tropea et al., 2009), it does not directly correct defects in neuronal migration or cortical layering. Therefore, once aberrant circuitry has been structurally consolidated, subsequent trophic stimulation may be insufficient to restore network‐level organization.

In addition, altered responsiveness of IGF‐1 signaling pathways in malformed cortex may contribute to treatment resistance. Disruptions in IGF‐1 receptor expression or downstream signaling cascades, such as the PI3K–Akt–mTOR pathway (D'Ercole and Ye, 2008; Littlejohn et al., 2021), could limit the capacity of exogenous (1–3)IGF‐1 to engage its intended molecular targets. Although these mechanisms were not directly assessed in the present study, they warrant investigation in future work.

Notably, prolonged exposure to (1–3)IGF‐1 resulted in measurable behavioral alterations, suggesting that the administered regimen was biologically active. This pattern raises the possibility that early and sustained trophic stimulation may shift excitatory–inhibitory balance in an already dysregulated network, consistent with an inverted U‐shaped dose–response relationship observed in cognitive modulation (Baldi and Bucherelli, 2005). Together, these findings suggest that developmental timing and circuit context may be critical determinants of IGF‐1 efficacy in malformed cortical systems.

Interestingly, behavioral findings raise further caution. (1–3)IGF‐1 could not affect the growth or structural integrity in malformed cortices (Figures 3 and 4). However, the impaired NP index in Y‐maze testing implies potential deficits in spatial working memory. Although contextual and cued fear recall were unaffected, increased freezing during the initial conditioning session may reflect altered emotional reactivity or sensory sensitivity (Fanselow, 1980) after long‐term (1–3)IGF‐1 treatment rather than enhanced learning (Kraeuter et al., 2019).

IGF‐1 is known to play a role in synaptic plasticity, spatial learning, and anxiety regulation. A previous animal study showed that IGF‐1 regulates intrinsic excitability and synaptic transmission in the infralimbic cortex, thereby facilitating the extinction of fear memory (Maglio et al., 2021). Moreover, recent studies (Harris, 2023; Keam, 2023) have demonstrated the promising efficacy of trofinetide, an (1–3)IGF‐1 analog, in improving core behavioral symptoms in patients with Rett syndrome, leading to its approval by the FDA. However, the dose–effect relationship of most biological drugs is not linear, but rather follows an inverted U‐shaped pattern—particularly with respect to cognitive function (Baldi and Bucherelli, 2005). Therefore, alternative dosing strategies involving different timings and durations should be considered to optimize the therapeutic potential of IGF‐1 treatment in MCD. Additionally, different disease entities may require distinct dosage regimens and treatment windows depending on their developmental trajectories, highlighting the need for more refined, individualized therapeutic plans.

Further, IGF‐1 treatment regimens used in this study had no impact on neurometabolic profiles of cingulate cortex at P15 (Figure 3B). Although the cingulate cortex is a cortical component of the limbic system and serves as a critical hub integrating emotional valence with cognitive control and autonomic regulation, the emotional or memory‐related changes observed in animals treated with (1–3)IGF‐1 may primarily involve other limbic structures, such as the hippocampus and amygdala (Kraeuter et al., 2019; LeDoux, 2000).

This study has several limitations that warrant consideration. First, outcome measures were assessed at only two developmental time points (P15 and P40–43), limiting our ability to capture long‐term or delayed effects of IGF‐1‐based interventions. Future studies incorporating extended longitudinal follow‐up could provide deeper insights into the durability and progression of treatment outcomes. Second, our analyses focused primarily on gross imaging, behavioral, and neurometabolic outcomes. Investigating cellular and synaptic‐level changes—such as synapse density, dendritic spine morphology, and electrophysiological properties—may help clarify the mechanistic basis for IGF‐1's limited efficacy in malformed cortical circuits. Third, the dosing regimens employed in this study were based on previously published protocols and exploratory considerations rather than systematic titration. Thus, our results may not reflect the optimal therapeutic window or dose–response relationship, and alternative dosing strategies could yield different outcomes (Iughetti et al., 2025; Keam, 2023; Lee et al., 2023; Vahdatpour et al., 2016). Lastly, the mechanisms underlying resistance to IGF‐1‐based therapies in MCD remain to be fully elucidated. Future studies integrating molecular profiling approaches, including transcriptomic or proteomic analyses, together with assessments of IGF‐1 receptor signaling and network‐level alterations, will be essential to clarify the mechanisms underlying treatment resistance and to refine therapeutic strategies for MCD.

## Conclusion

Despite the neurotrophic promise of IGF‐1 and growing clinical interest in its analogs, neither prenatal nor prolonged IGF‐1‐based interventions improved seizure susceptibility or neurometabolic profiles in this MCD model, and prolonged exposure produced detrimental behavioral effects. These findings highlight the challenges of applying IGF‐1 therapies in malformed cortical circuits and emphasize the need for mechanistic studies and carefully optimized treatment windows before clinical translation.

## Author Contributions

**Minyoung Lee**: investigation, formal analysis, writing methodology, validation, visualization. **Eun‐Jin Kim**: investigation, formal analysis, validation. **Mi‐Sun Yum**: validation, conceptualization, writing – original draft, writing – review and editing, funding acquisition, project administration, supervision.

## Funding

This work was supported by the Basic Science Research Program through the National Research Foundation of Korea (NRF) funded by the Ministry of Education (NRF‐2021R1A2C1004471) and a grant (2025IF0020) from the Asan Institute for Life Sciences, Asan Medical Center, Seoul, Korea.

## Conflicts of Interest

The authors declare no conflicts of interest.

## Supporting information




**Supplementary Material**: brb371405‐sup‐0001‐TableS1.tif

## Data Availability

The datasets generated and analyzed in the current study are available from the corresponding author upon reasonable request.
