# Supplementary figures and images for: Prenatal (1–3)IGF‐1 Treatment Is Ineffective and Behaviorally Detrimental in a Rat Model of Cortical Malformation
Source: Brain Behav. 2026 Apr 16;16(4):e71405. doi: 10.1002/brb3.71405 (PMC13087530; doi:10.1002/brb3.71405)

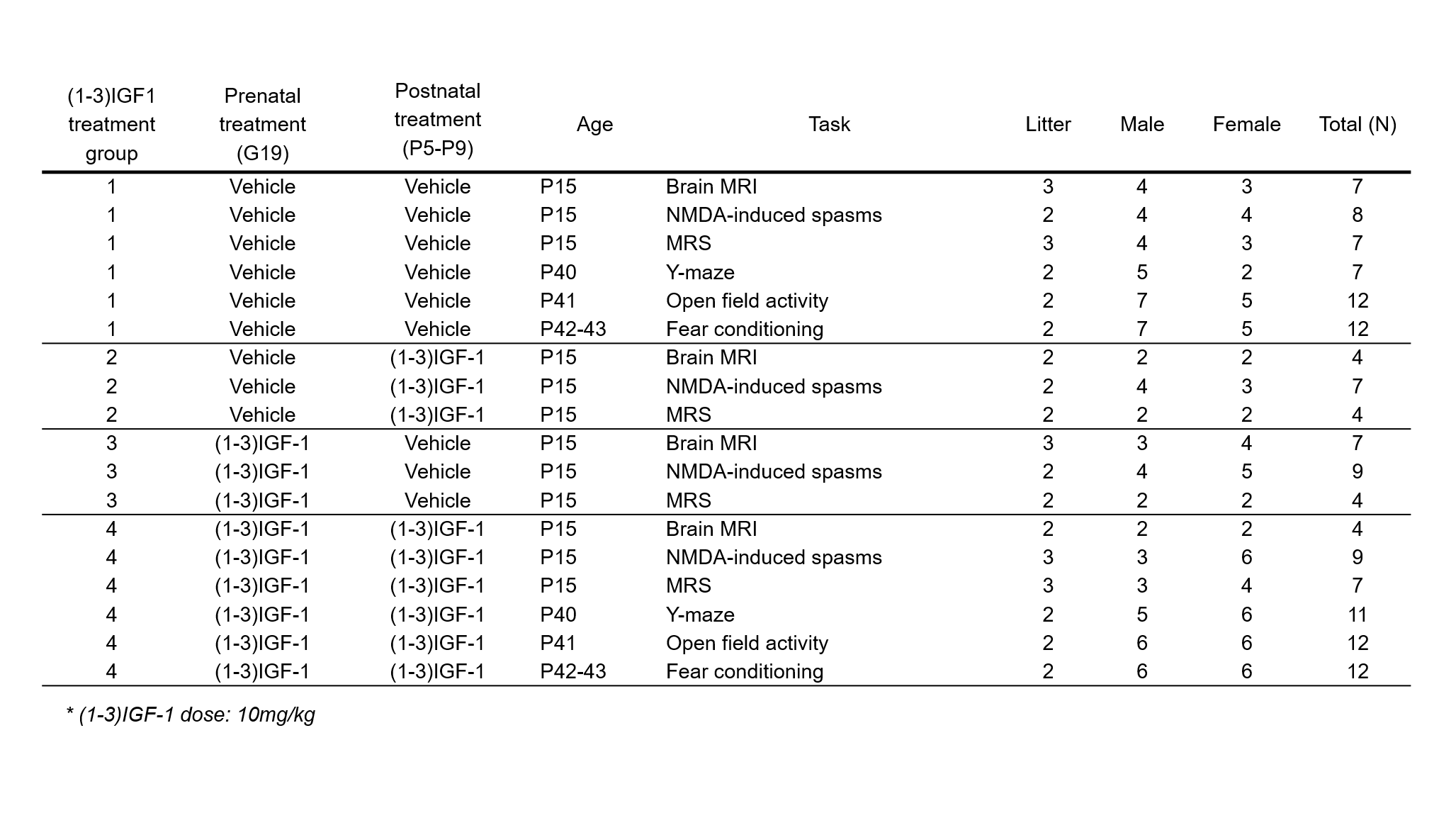

Supplement: Supplementary file 1 — Supplementary Material: brb371405‐sup‐0001‐TableS1.tif [file BRB3-16-e71405-s001.tif]
